# Mitigating cadmium accumulation in rice without compromising growth via modifying the regulatory region of *OsNRAMP5*

**DOI:** 10.1007/s44154-023-00117-x

**Published:** 2023-08-21

**Authors:** Zhenling Luo, Chao-Lei Liu, Xiaofei Yang, Jian-Kang Zhu, Chao-Feng Huang

**Affiliations:** 1 National Key Laboratory of Plant Molecular Genetics, Shanghai Center for Plant Stress Biology, Center for Excellence in Molecular Plant Sciences, Chinese Academy of Sciences, Shanghai, 200032 China; 2 University of the Chinese Academy of Sciences, Beijing, 100049 China; 3 State Key Laboratory of Rice Biology and Breeding, China National Rice Research Institute, Hangzhou, 310006 China; 4 Institute of Advanced Biotechnology and School of Life Sciences, Southern University of Science and Technology, Shenzhen, 518055 China

**Keywords:** Cadmium accumulation, CRISPR/Cas9, *OsNRAMP5* translation, *Oryza sativa* (rice), Regulatory region

## Abstract

Cadmium (Cd) intake poses a significant health risk to humans, and the contamination of rice grains with Cd is a major concern in regions where rice is a staple food. Although the knockout of *OsNRAMP5*, which encodes a key transporter responsible for Cd and manganese (Mn) uptake, can significantly reduce Cd accumulation in rice grains, recent studies have revealed that this knockout adversely affects plant growth, grain yield, and increases vulnerability to abiotic and biotic stresses due to reduced Mn accumulation. In this study, we employed CRISPR/Cas9 technology to modify the regulatory region of *OsNRAMP5* with the aim of reducing Cd accumulation in rice grains. Our findings demonstrate that mutations in the regulatory region of *OsNRAMP5* do not impact its expression pattern but result in a reduction in translation. The decreased translation of *OsNRAMP5* effectively decreases grain Cd accumulation while leaving Mn accumulation and important agronomic traits, including yield, unaffected. Thus, our study presents a practical and viable strategy for reducing Cd accumulation in rice grains without compromising Mn accumulation or overall rice production.

**Supplementary Information:**

The online version contains supplementary material available at 10.1007/s44154-023-00117-x.

The intake of cadmium (Cd) poses a serious health threat to humans, with rice being a major source of Cd intake for Asian people (Song et al. 2017; Tsukahara et al. 2003). In certain regions of southern China, soil contamination and acidification have led to a significant portion of rice grains surpassing the Chinese Cd limit (Zhao et al. 2015; Zhu et al. 2016). Consequently, there is an urgent need to reduce Cd accumulation in rice grains. Since Cd is not essential for plant growth, plants primarily transport Cd through manganese (Mn), iron (Fe), or zinc (Zn) transporters (Clemens et al. 2013). In rice, the OsNRAMP5 transporter, a member of the Natural Resistance-Associated Macrophage Protein (NRAMP) family, plays a crucial role in the uptake of both Mn and Cd uptake (Ishikawa et al. 2012; Sasaki et al. 2012). Knocking out *OsNRAMP5* significantly decreases Cd and Mn accumulation in shoots and grains. While earlier studies have suggested that *OsNRAMP5* knockout reduces Cd accumulation without affecting plant growth and grain yield (Ishikawa et al. 2012; Tang et al. 2017), recent reports have demonstrated that *OsNRAMP5* knockout lines exhibit diminished plant growth and grain yield, along with increased sensitivity to abiotic and biotic stresses due to reduced accumulation of the essential micronutrient Mn (Dong et al. 2021; Pei et al. 2023; Yang et al. 2019). These findings highlight the potential risk associated with reducing grain Cd accumulation through *OsNRAMP5* knockout for rice production. In this study, we present a feasible strategy to reduce grain Cd accumulation without affecting Mn accumulation and rice production by utilizing CRISPR/Cas9-mediated genome editing of the regulatory region of *OsNRAMP5*.

We designed five small guide RNAs to target specific regions of *OsNRAMP5*, located before the start codon (Fig. 1a). We obtained two lines, namely #17 and #27, which exhibited different deletion and insertion mutations within the regulator region of *OsNRAMP5* (Fig. 1a). Line #17 displayed a 166-bp deletion spanning -310 bp to -144 bp, along with a 1-bp insertion at -417 bp from the start codon of *OsNRAMP5*. On the other hand, line #27 featured a 190-bp deletion between -323 bp and -133 bp, with a 1-bp insertion at -417 bp from the start codon (Fig. 1a). Analyzing mRNA expression demonstrated that the mutations in the regulator region of *OsNRAMP5* in these two lines did not significantly impact the expression level of *OsNRAMP5* (Fig. 1b). To investigate whether the mutations influence the tissue-specific expression pattern of *OsNRAMP5*, we amplified the upstream regulatory region of *OsNRAMP5*, located before the start codon, from both WT and line #17, and fused them with β-glucuronidase (GUS) reporter gene, generating the *pOsNRAMP5*^*WT*^*:GUS* and *pOsNRAMP5*^*#17*^*:GUS* constructs, respectively. These constructs were subsequently introduced into rice plants. We selected two independent transgenic lines of *pOsNRAMP5*^*WT*^*:GUS* or *pOsNRAMP5*^*#17*^*:GUS* with similar *GUS* gene expression levels (Fig. 1c). GUS staining analysis revealed that the *pOsNRAMP5*^*#17*^ mutation did not significantly alter the GUS expression pattern but reduced the GUS signal (Fig. 1d). These results suggest that the *pOsNRAMP5*^*#17*^ mutation might impair the translation efficiency of *GUS*.

**Fig. 1:**
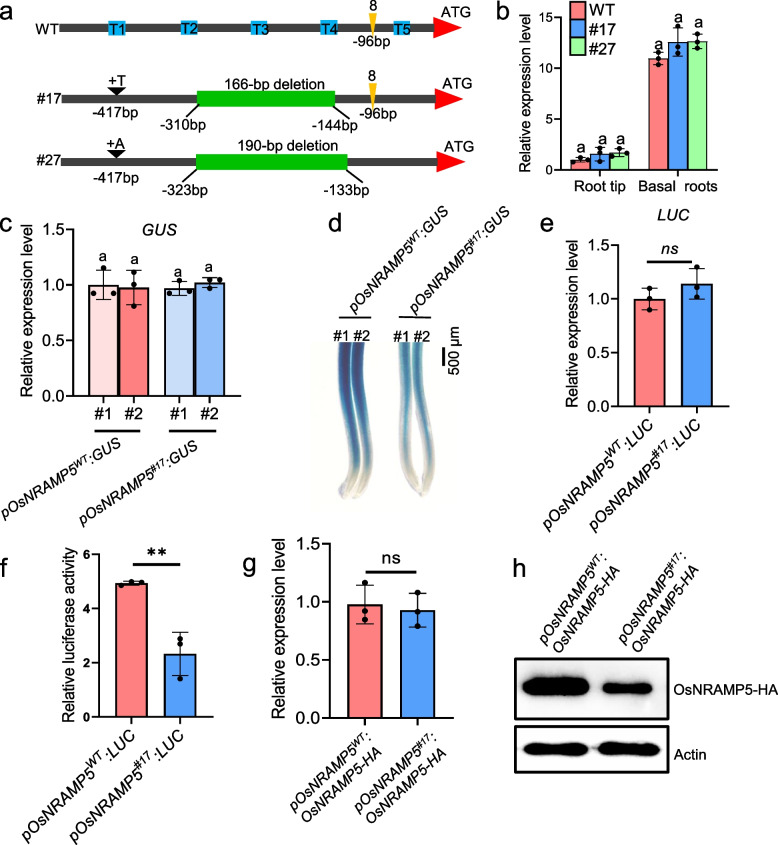
Mutations in the regulatory region of *OsNRAMP5* reduce OsNRAMP5 translation. **a** Diagram of mutations in the regulatory region of *OsNRAMP5* in two CRISPR/Cas9 lines (#17 and #27). Five guide RNAs (T1 to T5) indicated by blue boxes were designed for targeting the promoter of *OsNRAMP5*. Orange triangles indicate the 5’UTR in both WT and line #17, as revealed by 5’RACE data, while the numbers above the triangles correspond to the detected clone numbers. **b** Expression analysis of *OsNRAMP5* in WT and two lines with mutations in the regulatory region of *OsNRAMP5*. Root tips (0–1 cm) and basal roots (1–2 cm) of five-day-old seedlings were excised for the expression analysis. **c**, **d** Effect of *pOsNRAMP5*^*#17*^ mutation on *GUS* mRNA expression (**c**) and GUS activity (**d**) in rice. Two independent transgenic lines of both *pOsNRAMP5*^*WT*^*:GUS* and *pOsNRAMP*^*#17*^*:GUS* were subjected to mRNA expression analysis and GUS staining. Scale bar = 500 μm. **e**, **f** Effect of *pOsNRAMP5*^*#17*^ mutation on *LUC* expression (**e**) and LUC activity (**f**) in rice protoplasts. *pOsNRAMP5*^*WT*^*:LUC* or *pOsNRAMP*^*#17*^*:LUC* was co-expressed with *ZmUBQ:GUS* internal control in WT protoplasts, and then the protoplast were harvested for the *LUC* mRNA expression and LUC activity analyses. **g**, **h** Effect of *pOsNRAMP5*^*#17*^ mutation on mRNA expression (**g**) and protein accumulation (**h**) in rice protoplasts. Constructs of *pOsNRAMP5*^*WT*^*:OsNRAMP5-HA* and *pOsNRAMP5*^*#17*^*:OsNRAMP5-HA* were introduced into rice protoplasts for the mRNA expression and immunoblot analyses, respectively. Actin protein was used as an internal control. Data shown are means ± SD of three biological replicates. Means with different letters are significantly different (*P* < 0.05, ANOVA followed by Tukey test)

To further validate the negative effect of the *pOsNRAMP5*^*#17*^ mutation on translation, we fused the WT or mutated upstream regulatory regions of *OsNRAMP5* with luciferase reporter gene (*LUC*) and introduced the constructs, *pOsNRAMP5*^*WT*^*:LUC* and *pOsNRAMP5*^*#17*^*:LUC*, into rice protoplasts. The results showed that the *pOsNRAMP5*^*#17*^ mutation did not affect the mRNA expression of *LUC* but led to a decrease in the LUC signal (Fig. 1e and f). Additionally, we generated constructs of *pOsNRAMP5*^*WT*^*:OsNRAMP5-HA* and *pOsNRAMP5*^*#17*^*:OsNRAMP5-HA* and introduced them into rice protoplasts, respectively. Although the mRNA expression level of *OsNRAMP5-HA* was similar between the two constructs, there was reduced accumulation of OsNRAMP5-HA protein in the protoplasts expressing *pOsNRAMP5*^*#17*^*:OsNRAMP5-HA* compared to those expressing *pOsNRAMP5*^*WT*^*:OsNRAMP5-HA* (Fig. 1g and h). These findings collectively suggest that the *pOsNRAMP5*^*#17*^ mutation negatively impacts the translation of the target gene. It has been increasingly recognized that the 5’ untranslated region (5’UTR) of certain genes can adopt specific structures to modulate mRNA translation (Leppek et al. 2018). To examine whether the *pOsNRAMP5*^*#17*^ mutation affects the integrity of the 5’UTR of *OsNRAMP5*, we performed 5’ rapid amplification of cDNA ends (RACE) to identify the 5’UTR of *OsNRAMP5* in both the WT and line #17. However, our analysis only identified a 96-bp 5’UTR of *OsNRAMP5* (Fig. 1a), and no differences were found in the 5’UTR sequence in the WT and the mutant line. We suspect that the 5’UTR of *OsNRAMP5* may possess a complex secondary structure that prevents amplification by 5’RACE.

To evaluate the impact of the mutations in the regulatory region of *OsNRAMP5* on plant growth and metal accumulation, we conducted a growth experiment using WT, line #17, line #27, and an *Osnramp5* mutant control. The plants with two-weeks-old were grown in a nutrient solution containing 0.1 μM Cd and various concentrations of Mn for three weeks. Phenotypic analysis revealed that while the knockout mutant *Osnramp5* exhibited reduced plant growth and chlorophyll accumulation under low Mn conditions, the mutations in the regulatory region of *OsNRAMP5* did not affect plant growth or chlorophyll accumulation across all Mn conditions (Fig. 2a-d). Analysis of Cd concentration demonstrated no difference in root Cd accumulation between WT and the two lines with mutations in the regulatory region (Fig. 2f), although the *Osnramp5* mutant control exhibited significantly decreased Cd accumulation in the roots. However, the mutations in the regulatory region resulted in reduced Cd accumulation in the shoots at all Mn concentrations (Fig. 2e), albeit to a lesser extent than observed in the *Osnramp5* mutant. Furthermore, we assessed Mn concentration and observed that the mutations in the regulatory region did not influence Mn accumulation in either the roots or shoots under any Mn conditions (Fig. 2g and h), which suggest that other transporters may compensate for the impaired Mn transport caused by the mutations in the regulatory region of *OsNRAMP5*.

**Fig. 2:**
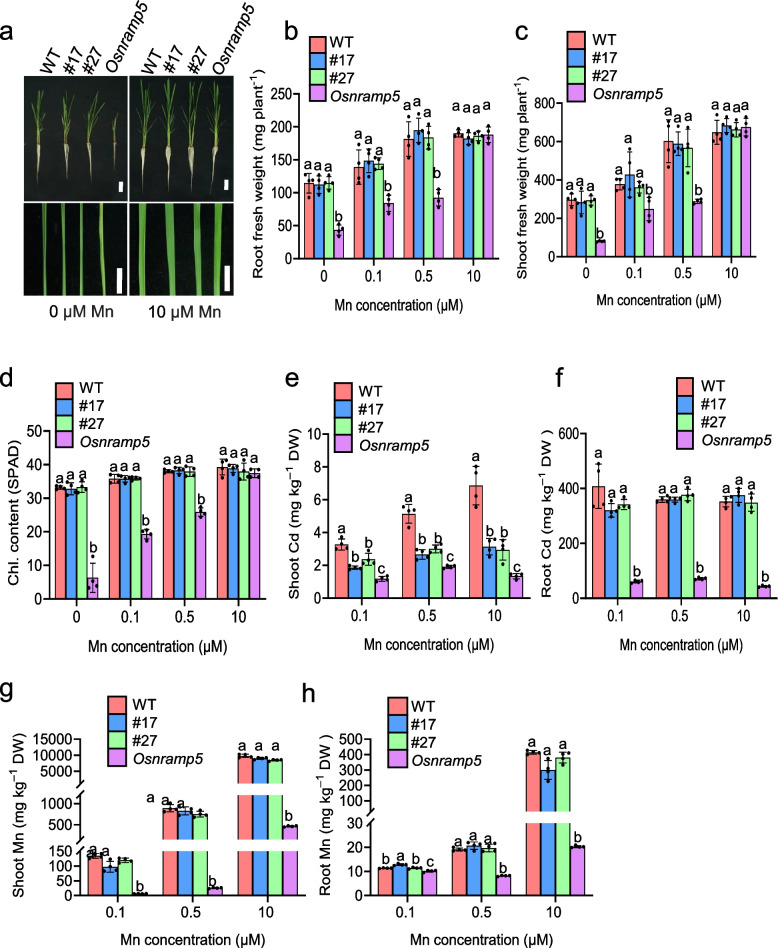
Mutations in the regulatory region of *OsNRAMP5* reduce shoot Cd accumulation under different Mn conditions. **a** Representative pictures of plant growth (upper panel) and leaf color (lower panel) of WT, line #17, line #27, and *Osnramp5* at 0 and 10 μM Mn. **b**-**d** Comparison of root (**b**) and shoot (**c**) fresh weight, and leaf chlorophyll content (**d**) of WT, line #17, line #27, and *Osnramp5* exposed to 0, 0.1, 0.5, or 10 μM Mn. SPAD values were used to calculate the chlorophyll content. **e**–**h** Effect of mutations in the regulatory region of *OsNRAMP5* on Cd accumulation in the roots (**e**) and shoots (**f**) and Mn accumulation in the roots (**g**) and shoots (**h**). Seedlings of WT, line #17, line #27, and *Osnramp5* were exposed to 0.1 μM Cd and 0.1, 0.5 or 10 μM Mn for three weeks. Cd and Mn concentrations in the roots and/or shoots were determined and compared. Data shown are means ± SD of four biological replicates. Means with different letters are significantly different (*P* < 0.05, ANOVA followed by Tukey test)

To assess the influence of the mutations in the regulatory region of *OsNRAMP5* on plant growth and grain Cd accumulation in real-world fields, we conducted a field study in a Cd-contaminated paddy field, where the soil had a Cd content of 1.25 ± 0.08 mg Kg^−1^ (*n* = 3) and a pH of was 5.72 ± 0.12 (*n* = 3). Analysis of agronomic traits revealed no significant differences in all measured parameters between the WT and the two lines with mutations in the regulatory region of *OsNRAMP5* (Fig. 3a-h). In contrast, the *Osnramp5* mutant control exhibited reduced plant height, panicle length, seed setting rate, and grain yield.

**Fig. 3:**
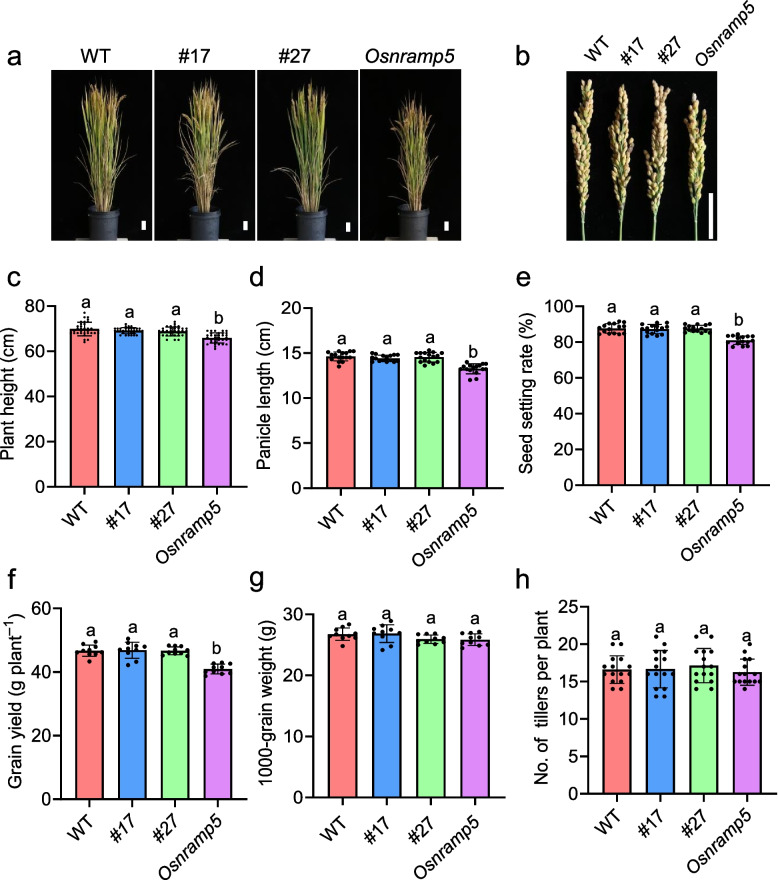
Analysis of agronomic traits in WT, line #17, line #27, and *Osnramp5* grown in paddy fields. **a**, **b** Images of the whole plant and panicle morphology at maturity period. Scale bars = 5 cm. **c** Plant height. Data are means ± SD (*n* = 33). **d** Panicle length. Data are means ± SD (*n* = 16). **e** Seed setting rate. Data are means ± SD (*n* = 10). **f** Grain yield. Data are means ± SD (*n* = 10).** g** Weight of 1000 grains. Data are means ± SD (*n* = 10). **h** No. of tillers per plant. Data are means ± SD (*n* = 15). Means with different letters are significantly different (*P* < 0.05, ANOVA followed by Tukey test)

We also measured the grain Cd content and found that the WT had a Cd content of 0.33 mg Kg^−1^, exceeding the Chinese Cd limit (0.2 mg Kg^−1^). However, the grain Cd content in line #17 and line #27 were 62% and 48% of that in WT, amounting to 0.20 and 0.16 mg Kg^−1^, respectively (Fig. 4a). The grain Mn content was similar between the WT and the two lines with mutations in the regulatory region of *OsNRAMP5* (Fig. 4b).

**Fig. 4:**
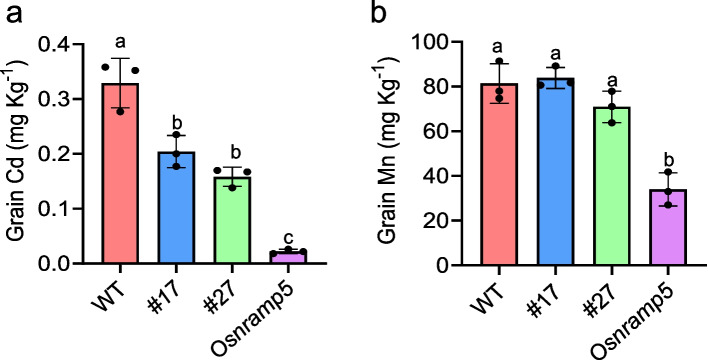
Mutations in the regulatory region of *OsNRAMP5* reduce grain Cd accumulation. **a**, **b** Grains of WT, line #17, line #27, and *Osnramp5* grown in paddy fields were sampled for the determination of Cd (**a**) and Mn (**b**) content. Data shown are means ± SD of three biological replicates. Means with different letters are significantly different (*P* < 0.05, ANOVA followed by Tukey test)

In summary, our findings demonstrate that mutations in the regulatory region of *OsNRAMP5* achieved through the CRISPR/Cas9 technology can reduce OsNRAMP5 translation, leading to a decrease in rice grain Cd accumulation without adversely affecting Mn accumulation and rice production.

## Materials and methods

### Plant materials

The wild-type (WT) controls used in this study had the genetic background of the japonica variety NanJing 46 (NJ46). The knockout mutant *Osnramp5*, which was previously identified and described by Yang et al. (2019), was used. To generate lines with mutations in upstream regulatory region of *OsNRAMP5*, we designed five small guide RNAs (Table S1) targeting specific regions within the upstream regulatory region of *OsNRAMP5*. These guide RNAs were then constructed them into a sgRNA-Cas9 expression vector. The resultant vector was transformed into NJ46 variety through *Agrobacterium* (EHA105 strain)*-*mediated transformation method. Two homozygous transgenic lines with different mutations in the upstream regulatory region (#17 and #27) were obtained and used for further experiments.

### Hydroponic experiments

Seeds of WT, line #17, line #27, and *Osnramp5* were soaked in water at 37 ℃ for 2 days and then transferred to a net floating on a 0.5 mM CaCl_2_ solution at 25℃. After two weeks, the plants were transferred to a half-strength Kimura B nutrient solution containing 0, 0.1, 0.5, or 10 μM MnSO_4_ and 0.1 μM CdCl_2_. The pH value of the nutrient solution was adjusted to 5.5, and the solution was renewed every 3 days. The plants were grown in an artificial climate chamber with a 12 h light period at 28℃ and a 12 h dark period at 22℃. After 3 weeks, the different genotypes were photographed and compared, and then the shoots and roots of the plants were sampled for the measurement of fresh weight.

### Field experiments

WT, line #17, line #27, and *Osnramp5* were cultivated in a Cd-contaminated paddy field with three plot replications in Fuyang, Hangzhou, China. The soil in this paddy field mainly consists of clay particles. The presence of excess Cd in the soil may be attributed to the previous use of Cd-polluted irrigation water, which was produced by a nearby papermaking factory. Each line in one plot was cultivated in six rows, with a total of ~ 36 plants. The plant growth in the field followed local cultivation habits, with water drained during the late tillering stage and grain filling stages. After grain heads were mature and ready to be harvested, agronomic traits including plant height, panicle length, number of tillers per plant, seed setting rate, 1000-grain weight, and grain yield were determined. Grains were sampled for the determination of metal content.

### Determination of metal content in plant tissues

Plant tissues, including roots, shoots, and grains, were dried at 65 °C for 7 days. A portion of the dried samples was weighted and digested with concentrated HNO_3_/HClO_4_ (85:15, v/v), followed by heating at 90℃ for 1 h and 105℃ for 3 h. The digested solution was then diluted to 10 mL with ultrapure water. The concentrations of Mn and Cd in the solution were determined using Inductively Coupled Plasma Mass Spectrometry (ICP-MS; PerkinElmer NexION300D).

### Determination of soil pH and Cd content

The air-dried soil was finely ground and mixed with water at a ratio of 2.5:1. The pH of the soil solution was measured using a pH meter. To determine the soil Cd content, 0.1 g of air-dried soil filtered with a 100-mesh sieve was soaked in a 2 mL mixed acid solution (HCl:HNO_3_ = 4:1, v/v) overnight. The sample was then digested at 35 °C for 3 h, 60 °C for 3 h, 105 °C for 1 h, and 120 °C for 2 h. After an overnight incubation, the samples were further digested with an additional 2 mL 20% HNO_3_ for 30 min at 80 °C and then fixed to 10 mL with ultrapure water. The concentration of Cd in the solution was determined using ICP-MS.

### RNA isolation and real-time RT–PCR analysis

Total RNA was extracted using the TRIzol reagent (Beyotime) and treated with DNase I to remove contaminating DNA. Approximately 1 μg of total RNA was used for first-strand cDNA synthesis with the HiScript 1st Strand cDNA Synthesis Kit (Vazyme Biotech, Nanjing, China). One-eighth of the resulting cDNA was used for real-time RT–PCR analysis with 2 × Universal SYBR Green Fast qPCR Mix (ABclonal, Wuhan, China). *Histone H3* (Os06g0130900) was used as an internal control for sample normalization (reference gene). Real-time RT-PCR was performed on the CFX96 Touch real-time PCR detection system (BioRad). The sequence information of the primers used for expression analysis is listed in Table S1.

### Identification of 5’UTR of OsNRAMP5

To determine the 5’UTR of *OsNRAMP5* in both the WT and line #17, 5′-RACE was performed with a HiScript-TS 5′/3′ RACE Kit (RA101; Vazyme, China) using 2 μg of total RNA. The ligated mRNA with RNA oligo adapter was used to synthesize first-strand cDNA using Template-Switching RT and random primers in a reverse transcription reaction. The 5′ ends of *OsNRAMP5* cDNA were amplified with primer 5’GSP (Table S1). The forward primer was conformed to the HiScript-TS 5′/3′ RACE Kit. PCR products were amplified by 2 × Phanta Max Master Mix (P525; Vazyme, China) DNA polymerase. The PCR conditions were as follows: 95 °C for 2 min, followed by 32 cycles at 95 °C for 20 s, the annealing temperature of 58 °C for 30 s, and the elongation temperature of 72 °C for 1 min. PCR products were gel purified with the Molpure Gel Extraction Kit (Yeasen, China) and cloned into pCE2 TA/Blunt-Zero vector (#C601; Vazyme, China) for sequencing.

### GUS analysis

To generate *pOsNRAMP5*^*WT*^*:GUS and pOsNRAMP5*^*#17*^*:GUS* transgenic lines, a primer pair (Table S1) was used to amply a 2.06-kb and 1.90-kb DNA fragment upstream of the start codon of *OsNRAMP5* from WT and a line #17, respectively. Each resulting DNA fragment was then fused with the *GUS* gene in the pCAMBIA1300 vector. The construct was introduced into WT plants by *Agrobacterium*-mediated transformation. Germinated seeds were grown in a solution with a 0.5 mM CaCl_2_ concentration for 2 days at pH 4.8. The roots were then stained with a commercialized GUS staining solution (161031; o'Biolab Co., Ltd., Beijing, China) for 15 min to 2 h at 37℃. Stained tissues were observed and photographed using a stereomicroscope (SZX12, Olympus) equipped with a camera (DP20, Olympus).

### Transient expression in protoplasts

To isolate protoplasts from young rice seedling, shoots were sliced into 1 mm strips and immersed in W5 solution (pH 5.7). The W5 solution consisted of 154 mM NaCl, 125 mM CaCl_2_, 5 mM KCl, and 2 mM 2-(N-morpholino) ethanesulfonic acid (MES). The strips were then digested in enzyme solution (pH 5.7) containing 1.2% Cellulase R-10, 0.6% Macerozyme R-10, 0.6 M mannitol, 0.1% BSA, and 10 mM MES. The digested solution was shaken for 4–5 h at 28 °C and filtered through a 145 μm mesh. The filtrate was diluted with five volumes of W5 solution and centrifuged at 150 g for 8 min to obtain the protoplasts. The protoplasts was resuspended in a suspension buffer composed of 0.6 M mannitol, 20 mM CaCl_2_ and 5 mM MES.

To examine the effect of the *pOsNRAMP5*^*#17*^ mutation on reporter gene translation, the ~ 2.0-kb DNA fragment upstream of the start codon of *OsNRAMP5* from WT and line #17 was amplified and fused with the *LUC* reporter gene to generate *pOsNRAMP5*^*WT*^*:LUC* and *pOsNRAMP5*^*#17*^*:LUC* constructs, respectively. Approximately 0.2 mL of rice protoplasts was co-transfected with 8 μg of *pOsNRAMP5*^*WT*^*:LUC* or *pOsNRAMP5*^*#17*^*:LUC* and 2 μg of *ZmUBQ:GUS* internal control. After overnight incubation, the protoplasts were collected by centrifugation at 150 g for 8 min. The collected protoplasts were then incubated with Firefly Luciferase Reporter Gene Assay Kit (RG005; Beyotime, Shanghai, China) for LUC signal detection or with 4-Methylumbelliferyl-*β-*D-Glucuronide for GUS signal detection.

To investigate the effect of the *pOsNRAMP5*^*#17*^ mutation on OsNRAMP5 accumulation, a DNA fragment consisting of ~ 2.0-kb DNA fragment upstream of the start codon and the gene of *OsNRAMP5* without a stop codon was amplified from WT or line #17 and cloned in frame with a 3HA tag into the pCAMBIA3301 vector. Approximately 0.4 mL of rice protoplasts was transfected with 15 μg of *pOsNRAMP5*^*WT*^*:OsNRAMP5-HA* or 15 μg of *pOsNRAMP5*^*#17*^*:OsNRAMP5-HA.* Total proteins were extracted using protein extraction buffer composed of SDS Lysis Buffer (Beyotime), 50 mM MG132, 100 mM Phenylmethanesulfonyl fluoride and 1 × Complete Protease inhibitor tablets EDTA-free (5892791001, Roche). Standard immunoblot analysis was performed to detect OsNRAMP5-HA using an anti-HA-HRP antibody (12013819001, Lot 44323100; Roche). The actin protein was used as a loading control and was detected using an anti-actin antibody (CW0264M; CoWin Biosciences, China).

### Supplementary Information

**Additional file 1: Table S1.** Primers used in this study.

## Data Availability

All data generated or analyzed during this study are included in this article.

## Abbreviations

Cd: Cadmium
Mn: Manganese
NRAMP: Natural Resistance-Associated Macrophage Protein
CRISPR: Clusetered regularly interspaced short plaindromic repeats
GUS: β-Glucuronidase
LUC: Luciferase
UTR: Untranslated region
RACE: Rapid amplification of cDNA ends
ICP-MS: Inductively Coupled Plasma Mass Spectrometry

## References

[CR1] Clemens S, Aarts MGM, Thomine S, Verbruggen N (2013). Plant science: the key to preventing slow cadmium poisoning. Trends Plant Sci.

[CR2] Dong J, Wu T, Sun Y, He H, Li Y, Peng Y, Ji Z, Meng Q, Zhao B, Tang L (2021). Effects of *OsNRAMP5* mutation on heat tolerance and main economic traits of rice under the conditions of different manganese concentration. Hybrid Rice.

[CR3] Ishikawa S, Ishimaru Y, Igura M, Kuramata M, Abe T, Senoura T, Hase Y, Arao T, Nishizawa NK, Nakanishi H (2012). Ion-beam irradiation, gene identification, and marker-assisted breeding in the development of low-cadmium rice. Proc Natl Acad Sci USA.

[CR4] Leppek K, Das R, Barna M (2018). Functional 5 ’ UTR mRNA structures in eukaryotic translation regulation and how to find them. Nat Rev Mol Cell Biol.

[CR5] Pei F, Wang G, Gao P, Feng Z, Hu K, Chen Z, Chen H, Cui A, Zuo S (2023). Evaluation of new japonica rice lines with low cadmium accumulation and good quality generated by knocking out *OsNramp5*. Chin J Rice Sci.

[CR6] Sasaki A, Yamaji N, Yokosho K, Ma JF (2012). Nramp5 is a major transporter responsible for manganese and cadmium uptake in rice. Plant Cell.

[CR7] Song Y, Wang YBN, Mao WF, Sui HX, Yong L, Yang DJ, Jiang DG, Zhang L, Gong YY (2017). Dietary cadmium exposure assessment among the Chinese population. PLoS One.

[CR8] Tang L, Mao BG, Li YK, Lv QM, Zhang LP, Chen CY, He HJ, Wang WP, Zeng XF, Shao Y, Pan YL, Hu YY, Peng Y, Fu XQ, Li HQ, Xia ST, Zhao BR (2017). Knockout of *OsNramp5* using the CRISPR/Cas9 system produces low Cd-accumulating indica rice without compromising yield. Sci Rep.

[CR9] Tsukahara T, Ezaki T, Moriguchi J, Furuki K, Shimbo S, Matsuda-Inoguchi N, Ikeda M (2003). Rice as the most influential source of cadmium intake among general Japanese population. Sci Total Environ.

[CR10] Yang CH, Zhang Y, Huang CF (2019). Reduction in cadmium accumulation in japonica rice grains by CRISPR/Cas9-mediated editing of OsNRAMP5. J Integr Agr.

[CR11] Zhao FJ, Ma YB, Zhu YG, Tang Z, McGrath SP (2015). Soil contamination in China: current status and mitigation strategies. Environ Sci Technol.

[CR12] Zhu HH, Chen C, Xu C, Zhu QH, Huang DY (2016). Effects of soil acidification and liming on the phytoavailability of cadmium in paddy soils of central subtropical China. Environ Pollut.

