# Supplementary material for: Mitigating cadmium accumulation in rice without compromising growth via modifying the regulatory region of OsNRAMP5
Source: Stress Biol. 2023 Aug 21;3(1):34. doi: 10.1007/s44154-023-00117-x (PMC10441987; doi:10.1007/s44154-023-00117-x)
Supplement: Supplementary file 1 — Additional file 1: Table S1. Primers used in this study. [file 44154_2023_117_MOESM1_ESM.docx]

**Table S1 Primers used in this study**

| **Primer** | **Sequence** | **Usage** |
| --- | --- | --- |
| OsNRAMP5-q1F | CAGCAGCAGTAAGAGCAAGATG | Real-time RT-PCR of *OsNRAMP5* |
| OsNRAMP5-q1R | GTGCTCAGGAAGTACATGTTGAT |  |
| OsNRAMP5-q2F | CGTCGTCTACCTCACCATCA | Real-time RT-PCR of *OsNRAMP5-HA* |
| OsNRAMP5-q2R | GGGACGTCATAGGGATAGCC |  |
| GUS-qF | TCAGGAAGTGATGGAGCATCA | Real-time RT-PCR of *GUS* |
| GUS-qR | CGGCAATAACATACGGCGTG |  |
| LUC-qF | AAGAGATACGCCCTGGTTCC | Real-time RT-PCR of *LUC* |
| LUC-qR | CCGATAAATAACGCGCCCAA |  |
| OsHistoneH3-qF | GGTCAACTTGTTGATTCCCCTCT | Real-time RT-PCR of *OsHistoneH3* |
| OsHistoneH3-qR | AACCGCAAAATCCAAAGAACG |  |
| pOsNRAMP5:LUC-F | CCTGCAGGCTCTAGAGGATCCAACTCCCACAACTACTGGGCC | Vector construct of *OsNRAMP5:LUC* |
| pOsNRAMP5:LUC-R | ATGTTTTTGGCGTCTTCCATGGCCTTGGGAGCGGGATGTCGG |  |
| pOsNRAMP5:GUS-F | ACGACGGCCAGTGCCAAGCTTAACTCCCACAACTACTGGGCC | Vector construct of *OsNRAMP5:GUS* |
| pOsNRAMP5:GUS-R | TACCATGGTACCCGGGGATCCTGTTGCTTCCTCTCTTAGC |  |
| pOsNRAMP5:OsNRAMP5-HA-F | CTTGCTCCGTGGATCCTCTAGAAACTCCCACAACTACTGGGC | Vector construct of *pOsNRAMP5:OsNRAMP5-HA* |
| pOsNRAMP5:OsNRAMP5-HA-R | GAACATCGTATGGGTATCTAGACCTTGGGAGCGGGATGTCGG |  |
| T1 | AAACAAGCTGTGGCTCCCCT | small guide RNAs targeting regulatory regions of *OsNRAMP5* |
| T2 | GTGCCCATGGAGATGCCAAA |  |
| T3 | TGGGGGCCTCCATGTACGTA |  |
| T4 | AATTTGGCTGCATACTTGCA |  |
| T5 | AAGCTAGAGCTCAGGCTAGC |  |
| 5’GSP | TCCAAGATTAGCTGCTAGCG | 5’RACE |
